# Molecular Epidemiology of HIV-1 Subtype B Infection across Florida Reveals Few Large Superclusters with Metropolitan Origin

**DOI:** 10.1128/spectrum.01889-22

**Published:** 2022-10-12

**Authors:** Shannan N. Rich, Mattia C. F. Prosperi, Simon Dellicour, Bram Vrancken, Robert L. Cook, Emma C. Spencer, Marco Salemi, Carla Mavian

**Affiliations:** a Department of Epidemiology, College of Public Health and Health Professions & College of Medicine, University of Florida, Gainesville, Florida, USA; b Emerging Pathogens Institute, University of Florida, Gainesville, Florida, USA; c Spatial Epidemiology Lab (SpELL), Université Libre de Bruxelles, Brussels, Belgium; d Department of Microbiology, Immunology and Transplantation, Rega Institute, Laboratory for Clinical and Epidemiological Virology, KU Leuven-University of Leuven, Leuven, Belgium; e Florida Department of Health, Division of Disease Control and Health Protection, Bureau of Communicable Diseases, Tallahassee, Florida, USA; f Department of Pathology, Immunology, and Laboratory Medicine, College of Medicine, University of Florida, Gainesville, Florida, USA; Johns Hopkins Hospital

**Keywords:** HIV, phylodynamics, molecular epidemiology, infection clusters, molecular networks, HIV in southeastern United States, Ending the HIV Epidemic (EHE) plan, networks, transmission clusters

## Abstract

Florida is considered an epicenter of HIV in the United States. The U.S. federal plan for Ending the HIV Epidemic (EHE) within 10 years prioritizes seven of Florida’s 67 counties for intervention. We applied molecular epidemiology methods to characterize the HIV infection networks in the state and infer whether the results support the EHE. HIV sequences (*N* = 34,446) and associated clinical/demographic metadata of diagnosed people with HIV (PWH), during 2007 to 2017, were retrieved from the Florida Department of Health. HIV genetic networks were investigated using MicrobeTrace. Associates of clustering were identified through boosted logistic regression. Assortative trait mixing was also assessed. Bayesian phylogeographic methods were applied to evaluate evidence of imported HIV-1 lineages and illustrate spatiotemporal flows within Florida. We identified nine large clusters spanning all seven EHE counties but little evidence of external introductions, suggesting—in the absence of undersampling—an epidemic that evolved independently from the rest of the country or other external influences. Clusters were highly assortative by geography. Most of the sampled infections (82%) did not cluster with others in the state using standard molecular surveillance methods despite satisfactory sequence sampling in the state. The odds of being unclustered were higher among PWH in rural regions, and depending on demographics. A significant number of unclustered sequences were observed in counties omitted from EHE. The large number of missing sequence links may impact timely detection of emerging transmission clusters and ultimately hinder the success of EHE in Florida. Molecular epidemiology may help better understand infection dynamics at the population level and underlying disparities in disease transmission among subpopulations; however, there is also a continuous need to conduct ethical discussions to avoid possible harm of advanced methodologies to vulnerable groups, especially in the context of HIV stigmatization.

**IMPORTANCE** The large number of missing phylogenetic linkages in rural Florida counties and among women and Black persons with HIV may impact timely detection of ongoing and emerging transmission clusters and ultimately hinder the success of epidemic elimination goals in Florida.

## INTRODUCTION

Incidence of HIV has remained relatively stable in the United States in recent years (1). Nevertheless, new diagnoses are not homogeneously distributed across the United States and some regions are disproportionately affected more than others (2). In 2017, population rates of new HIV diagnoses were highest in the South, where the state of Florida had the highest number of new diagnoses (1). In 2019, the U.S. Department of Health and Human Services (DHHS) released the federal plan for Ending the HIV Epidemic (EHE) within 10 years, identifying 48 counties with high incidence of HIV diagnoses, including seven urban Florida counties (Broward, Duval, Hillsborough, Miami-Dade, Orange, Palm Beach, and Pinellas), for initial funding (3). The EHE plan comprises multiple strategic approaches to reduce new HIV infections by 90% in the next 10 years, including building the capacity to detect and respond to ongoing and emerging clusters of HIV infection (3). Molecular epidemiology techniques (e.g., phylogenetics and phylodynamics) applied to viral genomic data can be used to identify genetic transmission clusters to prioritize for intervention (4, 5). Previous phylodynamic studies have identified external lineage introductions that may respond to drug regimens differently (6), revealed hidden transmission chains (7), and detected rapidly growing clusters of public health concern (8, 9).

The application of molecular methods to characterize the origin, spread, and infection dynamics of HIV in Florida remains to be explored. Per the enhanced HIV/AIDS Reporting System (eHARS), as of the end of 2020, approximately 117,000 people with HIV (PWH) are living in Florida (10), a highly diverse state with frequent tourism and domestic and foreign relocation. The Florida Department of Health (FDOH) has been collecting partial HIV-1 polymerase (*pol*) sequences from surveillance laboratories to monitor antiretroviral resistance since 2007, with the aim of reaching greater than 60% of persons with diagnosed HIV per year having an analyzable HIV nucleotide sequence within 12 months of diagnosis. The objective of this study was to apply molecular epidemiology techniques to identify HIV-1 clusters with high infection rates, evaluate evidence of imported lineages from outside geographic regions, and explore the phylogeographic spread of the largest clusters. These results were used to consider how the EHE plan could be improved in Florida by the FDOH.

## RESULTS

### Study population characteristics.

**FIG 1:**
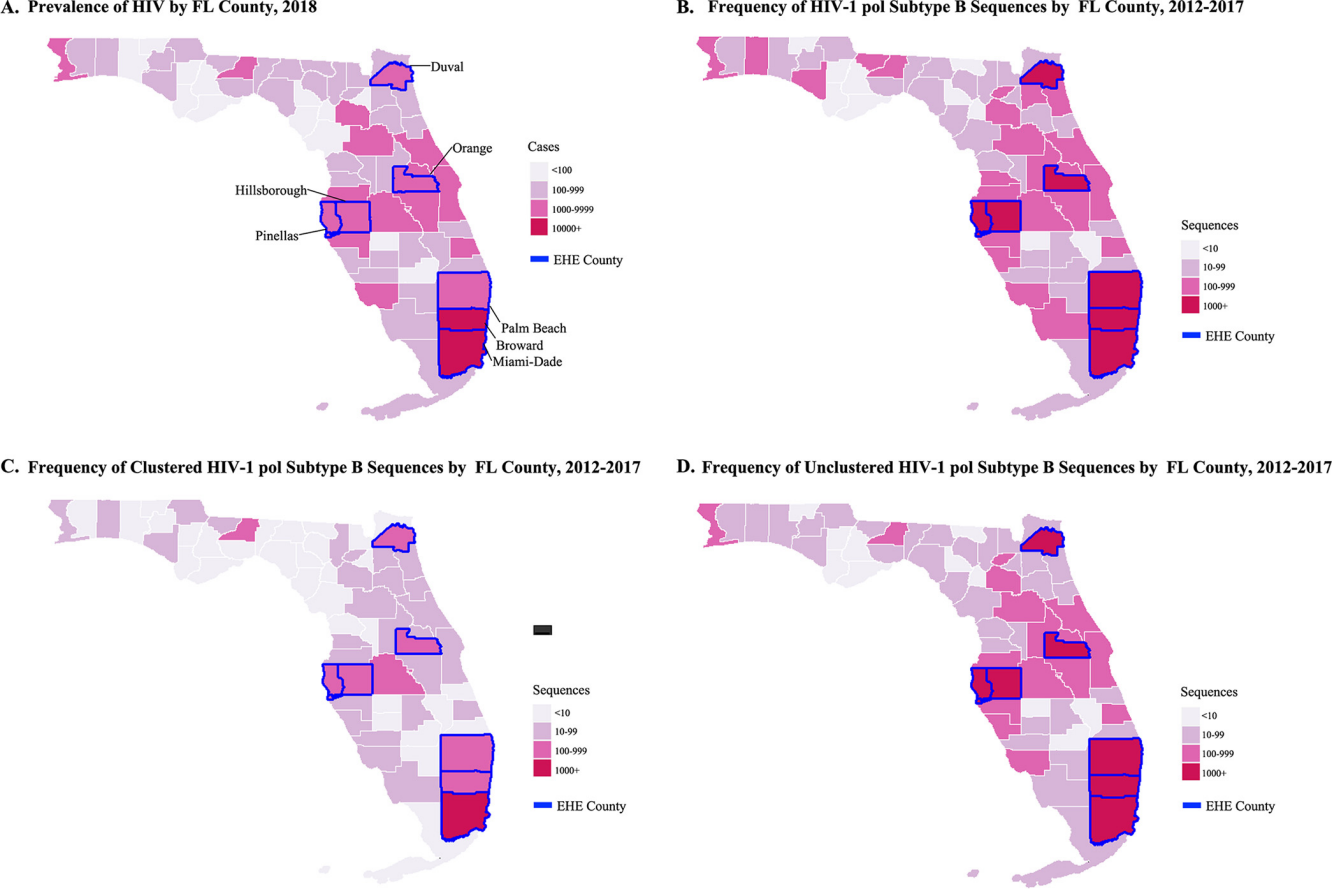
Geographic distribution of HIV-1 prevalence (A), frequency of HIV-1 subtype B polymerase (*pol*) sequences (B), frequency of HIV-1 subtype B *pol* sequences that clustered with at least one other sequence at a 1.5% pairwise genetic distance (C), and frequency of HIV-1 subtype B *pol* sequences that did not cluster (D) by Florida county. EHE, Ending the HIV Epidemic plan.

About 159,000 PWH were living in Florida during the study period of 2012 to 2017. Of these, between 46% and 91% did not have a genotype available for analysis, with the greatest differences seen between persons living in rural and urban locations (59.1% versus 84.6%, respectively). From eHARS, a total of 28,098 partial HIV-1 *pol* sequences from Florida collected during 2012 to 2017 and reported by July 2018 were considered for the transmission cluster analysis. Of these, 27,115 (96.5%) were classified as subtype B and included in subsequent analyses. Among these sequences, 4,943 (18.2%) clustered with at least one other, while the majority (81.8%) remained unclustered. During the sensitivity analysis, in which PWH with older diagnoses prior to 2010 were excluded (*n* = 14,640) and clusters were regenerated, the proportion of clustered sequences increased, as expected, to 32.1%; however, we observed a loss of 344 clusters and 936 PWH with older diagnoses that clustered with more recently diagnosed PWH (Table 1). Therefore, the full subtype B sequence data set collected 2012 to 2017 (*n* = 27,115) was retained for analysis. Most of the sequences originated from metropolitan regions containing the highest HIV prevalence (Fig. 1A and B), including all seven EHE priority counties: Miami-Dade (21.4%), Broward (18.0%), Palm Beach (6.4%), Duval (7.6%), Orange (8.3%), Pinellas (4.9%), and Hillsborough (6.7%). EHE priority counties also showed the highest proportion of clustered and unclustered sequences (Fig. 1C and D). Miami-Dade and Broward counties had the highest proportions of clustered sequences (21.6% and 16.4%, respectively) (Fig. 1), although the proportions of clustered sequences by county was not dependent on the number of sequences available (β coefficient = 7.217e-05, *P* = 0.232). Interestingly, several other counties, including rural and suburban counties with medium to high HIV prevalence, not currently considered an EHE priority, also showed a high proportion of unclustered sequences, indicating many missing infection links.

**TABLE 1:** Characteristics of persons with HIV-1 (PWH) who received a genotype in Florida during the 2012 to 2017 stratified-by-diagnosis period^*a*^

Characteristics	Population of PWH with genotype available during study period (2012 to 2017)^*b*^	Population of PWH without a genotype available during study period (2012 to 2017)	Cluster status of population with genotype during 2012 to 2017 (*N* = 27,115)			Cluster status of population with genotype during 2012 to 2017 diagnosed 2010 and beyond (*n* = 12,475)		
			Clustered (*n* = 4,943)	Unclustered (*n* = 22,172)	Clustered vs. unclustered aOR^*c*^ (95% CI^*d*^)	Clustered (*n* = 4,007)	Unclustered (*n* = 8,468)	Clustered vs. unclustered aOR (95% CI)
HIV Dx^*e*^ yr								
2016 to 17	5,146 (15.2%)	4,343 (2.6%)	1,426 (28.8%)	2,306 (10.4%)	Referent	1,323 (33.0%)	2,409 (28.4%)	Referent
2014 to 15	5,374 (15.9%)	3,848 (2.3%)	1,846 (37.3%)	2,720 (12.3%)	0.85 (0.76 to 0.95)	1,710 (42.7%)	2,856 (33.7%)	0.82 (0.73 to 0.94)
2012 to 13	2,788 (8.3%)	5,994 (3.6%)	815 (16.5%)	1,844 (8.3%)	0.48 (0.41 to 0.55)	731 (18.2%)	1,928 (22.8%)	0.45 (0.38 to 0.53)
2010 to 11	1,842 (5.5%)	7,452 (4.5%)	278 (5.6%)	1,240 (5.6%)	0.23 (0.19 to 0.27)	243 (6.1%)	1,275 (15.1%)	0.21 (0.18 to 0.26)
<2010	15,069 (44.6%)	144,339 (87.0%)	578 (11.7%)	14,062 (63.4%)	0.04 (0.04 to 0.05)			
Sampling yr								
Median (IQR^*f*^)	Not available	Not available	2015 (2014 to 16)	2015 (2014 to 16)	0.88 (0.85 to 0.91)	2015 (2014 to 16)	2015 (2014 to 16)	0.87 (0.83 to 0.91)
Birth region								
North America	21,869 (64.7%)	122,510 (73.8%)	3,781 (81.4%)	16,437 (77.6%)	Referent	3,014 (80.5%)	5,492 (70.1%)	Referent
Africa	122 (0.4%)	578 (0.3%)	6 (0.1%)	20 (0.1%)	0.77 (0.25 to 2.19)	5 (0.1%)	8 (0.1%)	0.68 (0.20 to 2.15)
Asia Pacific	168 (0.5%)	710 (0.4%)	28 (0.6%)	74 (0.3%)	0.82 (0.48 to 1.37)	26 (0.7%)	43 (0.5%)	0.93 (0.53 to 1.60)
Caribbean	4,836 (14.3%)	26,635 (16.0%)	536 (11.5%)	3,398 (16.0%)	0.63 (0.55 to 0.71)	441 (11.8%)	1,537 (19.6%)	0.65 (0.56 to 0.75)
Europe	187 (0.6%)	1,317 (0.8%)	33 (0.7%)	119 (0.6%)	0.56 (0.36 to 0.85)	29 (0.8%)	74 (0.9%)	0.53 (0.33 to 0.83)
Latin America	1,701 (5.0%)	8,640 (5.2%)	261 (5.6%)	1,135 (5.4%)	0.42 (0.35 to 0.50)	227 (6.1%)	685 (8.7%)	0.42 (0.35 to 0.51)
Age at genotyping (yr)								
Mean (*SD*^*g*^)	Not available	Not available	31.4 (11.5)	35.1 (12.2)	0.96 (0.95 to 0.96)	31.3 (11.3)	38.7 (13.3)	0.95 (0.95 to 0.95)
Sex at birth								
Female	9,096 (26.9%)	43,270 (26.1%)	765 (15.5%)	7,068 (31.9%)	Referent	542 (13.5%)	2,205 (26.0%)	Referent
Male	21,123 (62.5%)	122,706 (73.9%)	4,178 (84.5%)	15,104 (68.1%)	1.35 (1.19 to 1.53)	3,465 (86.5%)	6,263 (74.0%)	1.31 (1.13 to 1.53)
Race/ethnicity								
Black	16,556 (49.0%)	77,817 (46.9%)	2,354 (47.6%)	12,212 (55.1%)	Referent	1,826 (45.6%)	4,325 (51.1%)	Referent
Hispanic/Latino	6,511 (19.3%)	31,810 (19.2%)	1,215 (24.6%)	4,339 (19.6%)	1.32 (1.18 to 1.48)	1,044 (26.1%)	2,077 (24.5%)	1.38 (1.21 to 1.56)
Other	692 (2.0%)	3,293 (2.0%)	110 (2.2%)	462 (2.1%)	1.10 (0.83 to 1.44)	87 (2.2%)	156 (1.8%)	1.09 (0.79 to 1.50)
White	6,460 (19.1%)	53,056 (32.0%)	1,264 (25.6%)	5,159 (23.3%)	1.11 (1.01 to 1.23)	1,050 (26.2%)	1,910 (22.6%)	1.26 (1.12 to 1.41)
District								
Central East	3,694 (10.9%)	18,139 (10.9%)	734 (14.8%)	66 (12.5%)	Referent	592 (14.8%)	1,031 (12.2%)	Referent
Central West	4,890 (14.5%)	23,751 (14.3%)	854 (17.3%)	2,769 (16.6%)	0.80 (0.70 to 0.92)	725 (18.1%)	1,469 (17.3%)	0.86 (0.74 to 1.00)
Northeast	7,218 (21.4%)	14,896 (9.0%)	715 (14.5%)	2,804 (12.6%)	1.05 (0.91 to 1.22)	558 (13.9%)	1,030 (12.2%)	1.01 (0.85 to 1.20)
Northwest	1,634 (4.8%)	6,720 (4.0%)	282 (5.7%)	1,246 (5.6%)	0.86 (0.72 to 1.04)	226 (5.6%)	470 (5.6%)	0.87 (0.71 to 1.07)
Southeast	14,982 (44.3%)	93,646 (56.4%)	2,140 (43.3%)	10,633 (48.0%)	0.90 (0.80 to 1.00)	1,728 (43.1%)	4,074 (48.1%)	0.92 (0.81 to 1.05)
Southwest	1,369 (4.1%)	8,824 (5.3%)	207 (4.2%)	983 (4.4%)	0.88 (0.72 to 1.07)	170 (4.2%)	378 (4.5%)	0.91 (0.73 to 1.15)
Rural/urban								
Rural	4,759 (14.1%)	6,871 (4.1%)	118 (2.4%)	790 (3.6%)	Referent	93 (2.3%)	235 (2.8%)	Referent
Urban	29,028 (85.9%)	159,105 (95.9%)	4,814 (97.6%)	21,316 (96.4%)	1.42 (1.12 to 1.81)	3,906 (97.7%)	8,217 (97.2%)	1.24 (0.94 to 1.66)
Transmissioncategory								
HET^*h*^	11,284 (33.4%)	48,612 (29.3%)	1,064 (21.5%)	8,530 (38.5%)	Referent	768 (19.2%)	3,168 (37.4%)	Referent
IDU^*i*^	3,139 (9.3%)	27,835 (16.8%)	344 (7.0%)	2,830 (12.8%)	1.17 (1.00 to 1.37)	255 (6.4%)	546 (6.4%)	1.41 (1.16 to 1.72)
MSM^*j*^	13,939 (41.3%)	70,153 (42.3%)	3,377 (68.3%)	9,376 (42.3%)	1.56 (1.38 to 1.75)	2,870 (71.6%)	4,251 (50.2%)	1.68 (1.46 to 1.92)
MTC^*k*^	612 (1.8%)	1,901 (1.1%)	31 (0.6%)	525 (2.4%)	0.26 (0.17 to 0.38)	12 (0.3%)	58 (0.7%)	0.19 (0.09 to 0.35)
Unknown	1,245 (3.7%)	17,475 (10.5%)	127 (2.6%)	911 (4.1%)	0.84 (0.67 to 1.06)	102 (2.5%)	445 (5.3%)	0.90 (0.69 to 1.16)

a Descriptive results are presented as frequency (column percentage) unless otherwise specified. Missing values were retained as unknown for this table, and therefore, not all proportions and inverse proportions add up to 1.

b Sequences were available for 31.6% of the total population living with HIV using state prevalence data (flhealthcharts.com [Accessed 5 April 2020]).

c aOR, adjusted odds ratio.

d CI, confidence interval.

e Dx, diagnosis.

f IQR, interquartile range.

g *SD*, standard deviation.

h HET, heterosexual.

i IDU, intravenous drug use.

j MSM, men who have sex with men.

k MTC, mother-to-child.

### Characteristics of PWH with clustered versus unclustered sequences.

Both the clustered and unclustered populations were majority male (71.1%), Black (53.7%), and men who have sex with men (MSM, 47.0%) or heterosexual (HET, 35.4%) (Table 1). During the multivariable model selection phase, the same model retaining all variables was generated by both feature selection approaches; therefore, all variables were included in the final model. Compared with unclustered sequences, clustered sequences tended to be from PWH who were, on average, younger at genotype collection, male versus female, Hispanic/Latino versus Black, from a county of residence in the central east district of Florida versus the central west, from an urban versus rural county, and born in North America versus the Caribbean, Europe, or Latin America. Further, MSM had higher odds of clustering compared with persons with HET contact whereas persons with MTC transmission had lower clustering odds. Persons diagnosed in the most recent year (2016 to 2017) had greater odds of clustering compared with all other years. These findings were largely unaffected after removing persons with older diagnoses during the sensitivity analysis, indicating that cluster propensity was robust to diagnosis year (Table 1).

### Infection cluster features.

**FIG 2:**
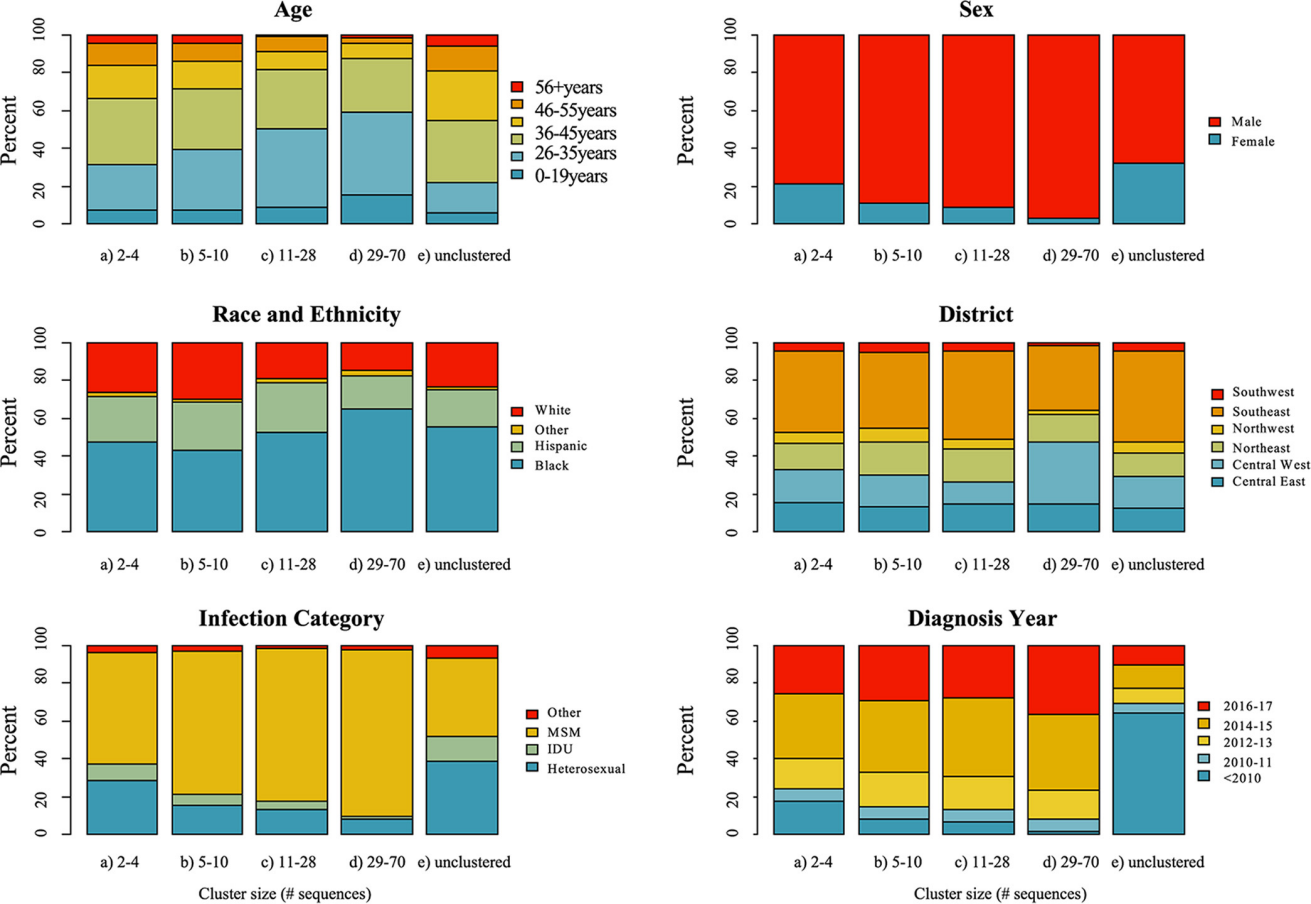
Clinical and demographic traits of Florida HIV-1 subtype B cluster members by cluster size. MSM, men who have sex with men; IDU, intravenous drug use; Other race, American Indian/Alaska Native, Asian, Native Hawaiian/Pacific Islander, or Multirace; Other transmission category, perinatal, occupational, or unknown.

Of the clustered sequences, 3,077 (57.4%) and 1,165 (21.7%) were clustered within the smallest clades containing two to four sequences and five to 10 sequences, respectively. A total of 778 (14.5%) sequences were in medium clusters (sized 11 to 28 sequences) and 339 (6.3%) in large clusters (sized 29 to 70). Most clustered sequences were from HIV diagnoses in more recent years (2014 to 2017), and this pattern was even more pronounced in the largest clusters (Fig. 2). Cluster size was inversely associated with the mean age of cluster members; PWH aged ≥36 years were more frequently linked in small clusters, while PWH aged ≤25 years represented the largest subpopulation in the largest clusters. The smallest clusters contained a high proportion of members that were female, heterosexual, and diagnosed with HIV before 2010. Compared with small and medium clusters, there was a higher proportion of members in the largest clusters that were male, Black, from a central-west county, born in North America, and MSM.

Assortative mixing, evidenced by assortativity coefficients [*r*]>0 (with *r *> 0.4 indicative of strong “likeness”), differed by cluster size (Table 2). Larger clusters containing 11 to 70 sequences were minimally assortative by age (range = 0.09 to 0.11), transmission category (range = 0.06 to 0.19), and sampling year (range = 0.09 to 0.11). Alternatively, smaller clusters, including those containing two to 10 sequences, were assortative by age (range = 0.36 to 0.38), transmission category (range = 0.38 to 0.46), and sampling year (range = 0.36 to 0.38). Assortativity was highest for geographic region, i.e., county (range = 0.32 to 0.58) and district (range = 0.48 to 0.75), and demographic for all cluster sizes.

**TABLE 2:** Assortativity coefficients of select attributes by cluster size, 2012 to 2017

Attribute	Cluster size (# sequences)			
	2 to 4	5 to 10	11 to 28	29 to 70
Assortativity coefficient (*r*)				
Demographics (age, gender, race/ethnicity, transmission group)^*a*^	0.23 to **0.48**^*b*^	0.38 to **0.48**	0.11 to **0.44**	0.06 to 0.36
County	**0.58**	**0.47**	**0.43**	0.32
District	**0.75**	**0.65**	**0.68**	**0.48**
Sampling yr	0.36	0.38	0.11	0.09

a Estimates are provided as ranges upon ethical considerations.

b Coefficients demonstrating the strongest relationships are in bold.

### Analysis of the largest clusters’ infection rates, origins, and within-state phylogeography.

**FIG 3:**
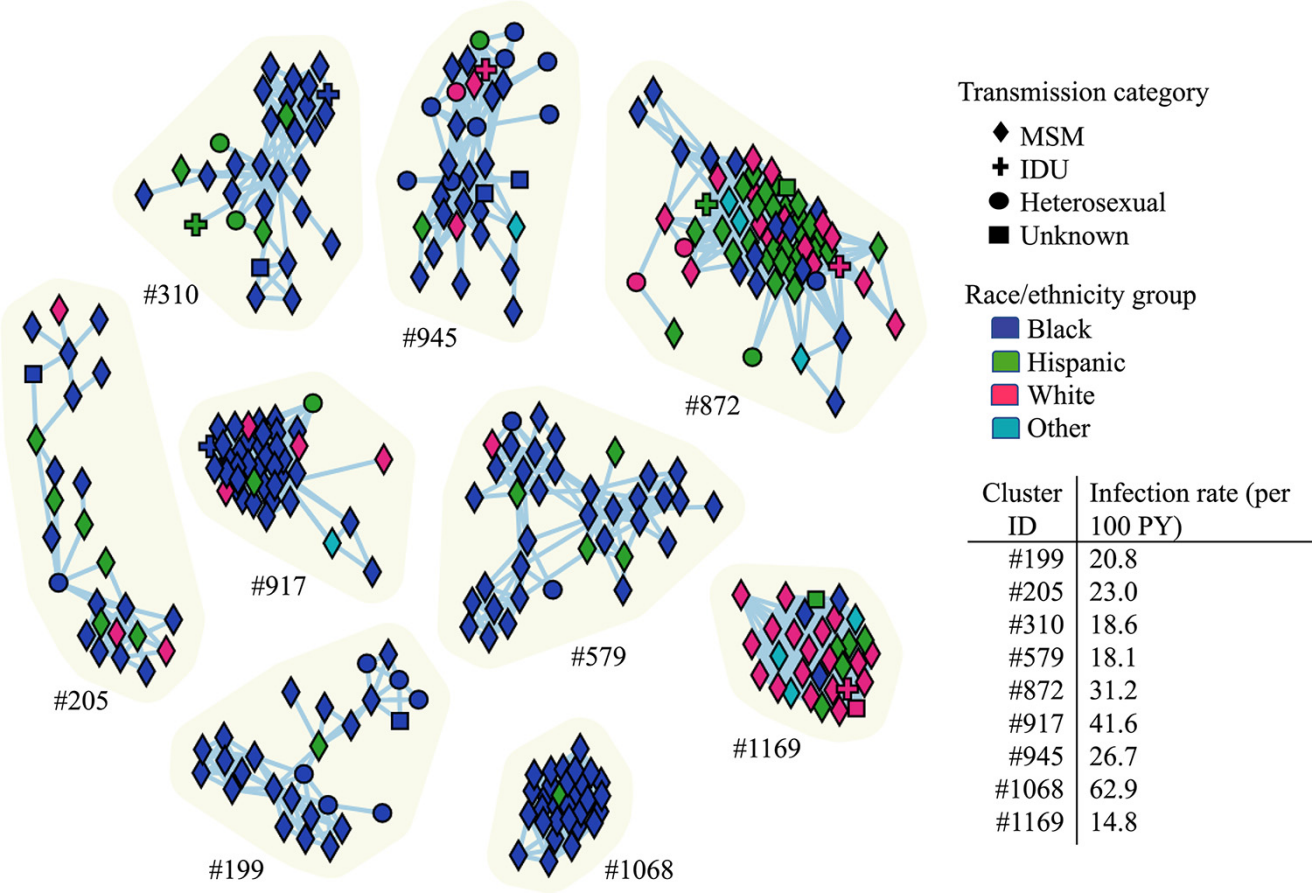
Infection networks of the largest HIV-1 subtype B clusters in Florida inferred using a genetic distance threshold of 1.5% in MicrobeTrace. Node shape corresponds to transmission category: diamond (MSM, men who have sex with men), plus sign (IDU, intravenous drug use), circle (HET, heterosexual contact), and square (unknown). Nodes are colored according to race/ethnicity group: blue (Black), green (Hispanic), pink (White), and turquoise (Other, includes American Indian/Alaska Native, Asian, Native Hawaiian/Pacific Islander, or Multirace individuals). Transmission rates reflect the number of transmissions observed per 100 person-years (PY).

**FIG 4:**
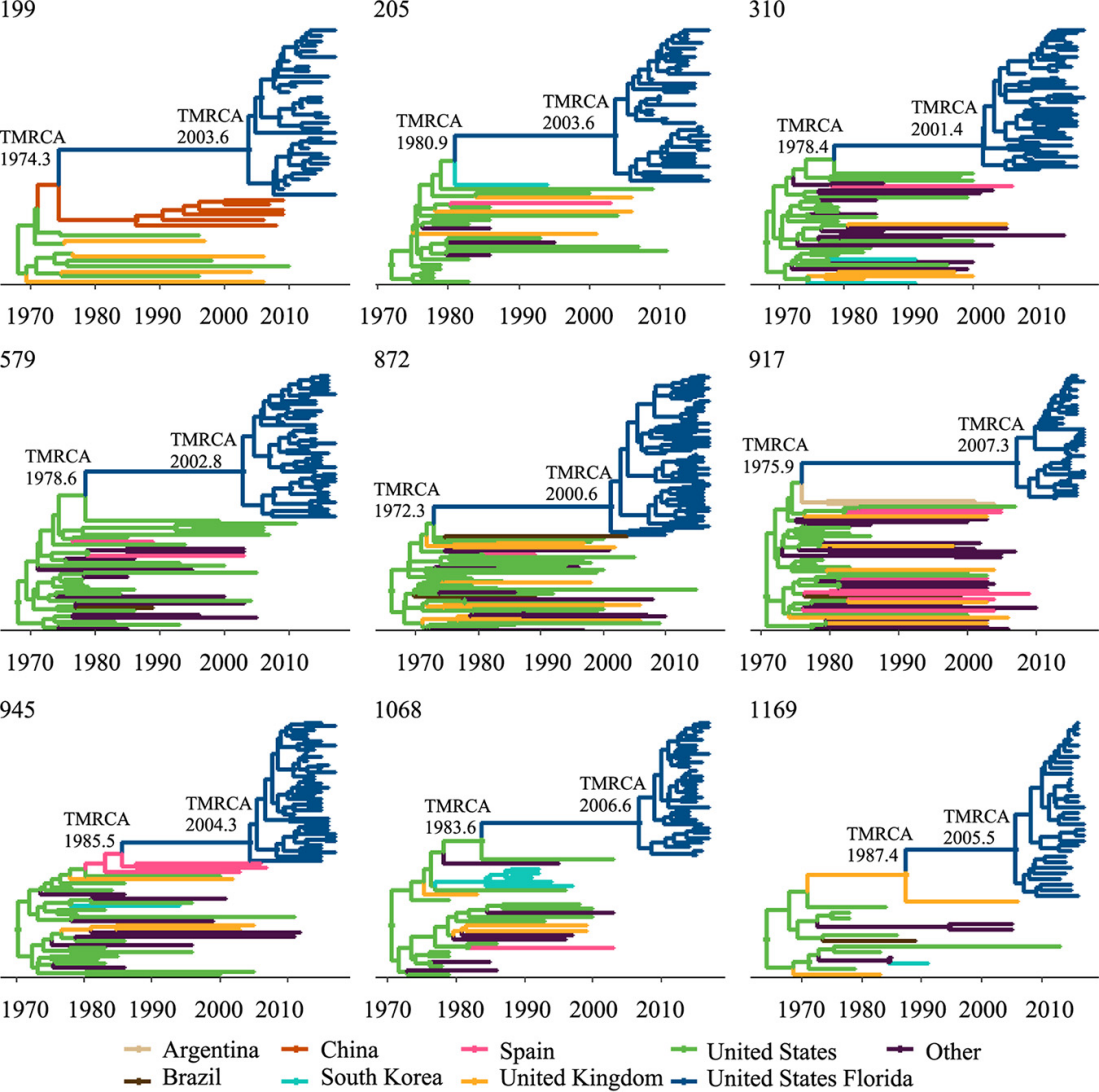
Bayesian phylodynamic reconstruction of the nine largest HIV-1 subtype B clusters in Florida with reference sequences from the Los Alamos National Laboratory (LANL) database. The maximum clade credibility (MCC) time-scaled phylogenies were inferred using the relaxed molecular clock and skyline demographic priors implemented in BEAST v1.10.4, and a discrete asymmetric trait analysis. Circles represent branches supported by posterior probability >0.90. Branches are colored based on location of origin as indicated in the key (e.g., the Florida sequences in cluster #1169 share an ancestor with a sequence from the United Kingdom). Time of the most recent common ancestor (TMRCA) for Florida clusters and for the coalescent event with the most recent ancestor from LANL are indicated at the respective nodes, for 95% HPD intervals see Table S2.

The largest clusters were predominantly composed of MSM; however, nearly all comprised more than one infection risk group (Fig. 3). Infection rates of the largest clusters ranged from 14.8 infections per 100 person-years (cluster #1169) to 62.9 per 100 person-years (cluster #1068), well over the national estimate of four infections per 100 person-years (11). The time to the most common ancestor (TMRCA) of the largest clusters dated between 2003 and 2012 (Fig. 4; Table S2). The coalescent event with the closest HIV sequence available in the LANL databases dated as far back as 1972.3 and for cluster #1169 was around 1987, close to the start of the HIV-1 epidemic in Florida (12). Most of the clustered sequences shared an ancestor with other sequences from the US (Fig. 4; Table S3), including those from individuals in California, Washington, Oregon, and one from the multi-U.S. city EXPLORE study cohort (13) (Table S3). We observed evidence of importation from South America (Brazil in cluster #199 and #872 and Argentina in #917), South Korea (cluster #205), and the United Kingdom (cluster #1169), although these introductions were dated several decades ago, suggesting that the source of the introduction has not been sampled yet (Table S3). Evaluation of linkages between a sample of unclustered Florida sequences with worldwide subtype B sequences available in public HIV databases did not yield significant improvement in clustering results. We observed only two (<1%) of the Florida sequences clustering with sequences available in the LANL database: one from the HIV Vaccine Trials Network (at 0.00981 distance), the other from the HIV EXPLORE Study (at 0.01458 distance).

**FIG 5:**
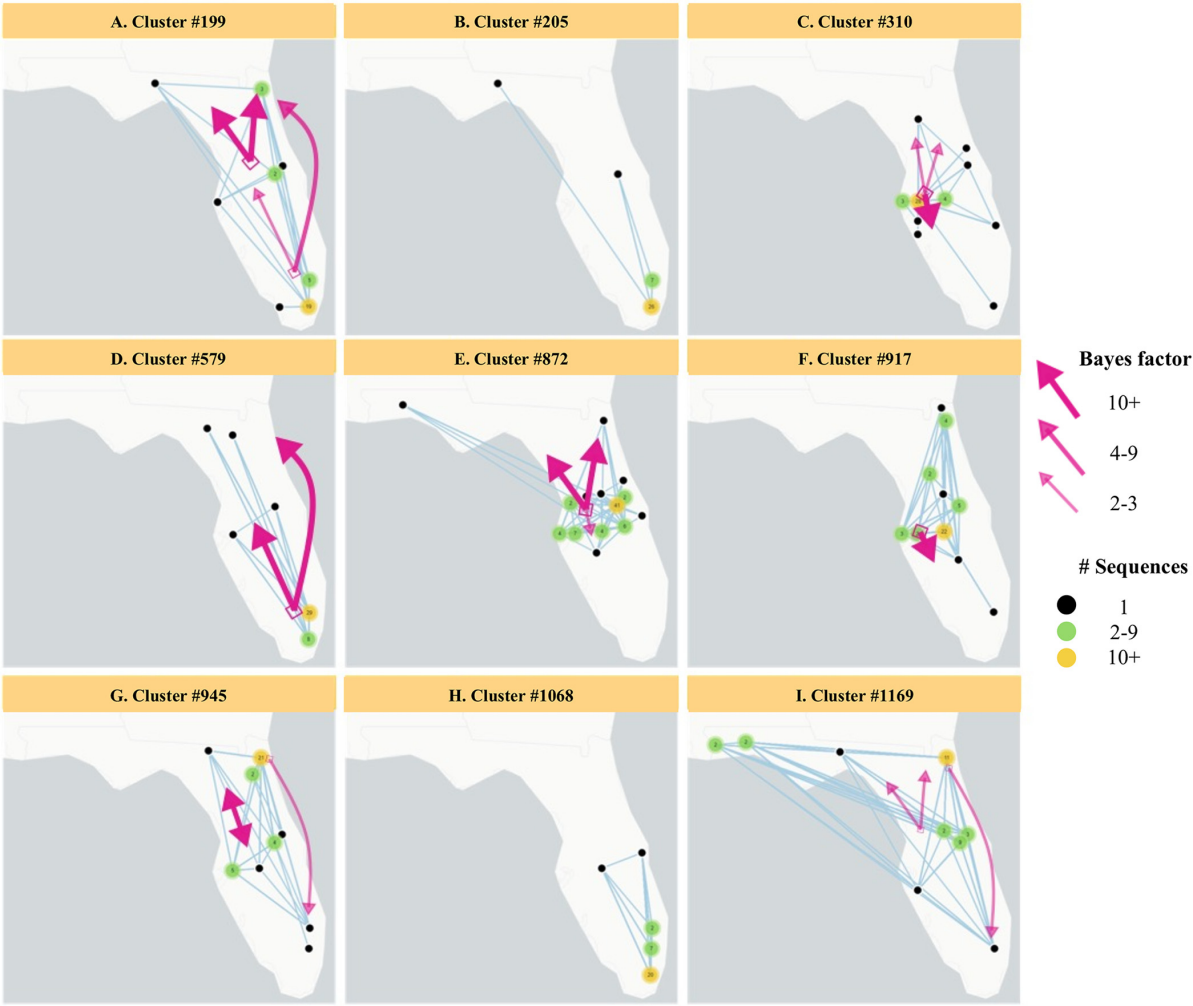
The largest HIV-1 subtype B clusters in Florida mapped by county, with arrows representing the rates of spatial-temporal diffusion between regions (north, central, and south) inferred by Bayesian analysis. Black dots represent counties with one sequence in a cluster. Green dots represent counties with two to nine sequences in a cluster. Yellow dots represent counties with >10 sequences in a cluster. Numbers in dots show actual number of sequences in the cluster. Arrow width and color correspond to the strength of evidence available for these diffusion rates, as indicated by the adjusted Bayes factors (Table S4). Patterns of migration from the south to north and south to central Florida were observed for cluster #199 and #579. Migration from central to north Florida was observed for cluster #199, #310, #872, #945, and #1169. Migration from central to south Florida was observed for clusters #310, #872, and #917. We also observed evidence of migration from north to central Florida (cluster #945) and weak evidence from the north to south regions (cluster #945 and #1169). The migration patterns for clusters #205 and #1068 are unknown as the results were not well supported after adjusting for sampling bias.

Spatiotemporal patterns showed that sequences from persons in the largest clusters often spanned multiple geographic locations, except for clusters #205 and #1068 for which most cluster members were from South Florida, including Miami-Dade, Broward, and Palm Beach counties that together represent a large metropolitan area (Fig. 5) but which were not well supported after adjusting for sampling bias (Table S4). The other seven large HIV clusters were observed crossing multiple neighboring and non-neighboring Florida counties, and in some instances, spanning the entire state (#199, #579, #945, #1169). The phylogeographic analysis, adjusted for location sampling bias, revealed strong evidence of the large HIV clusters deriving from the south and central regions of Florida and spreading to other regions (Table S4).

## DISCUSSION

We report, for the first time, an in-depth molecular epidemiology and spatiotemporal analysis of the HIV epidemic in Florida. We identified factors associated with infection cluster status and size, assessed cluster demographic features, and inferred the origin and putative geographic spread of the largest clusters across the state. Considering that Florida is a popular state for tourism and domestic and foreign relocation, we investigated whether the largest clusters were connected to recent introductions from other U.S. states or countries, as has previously occurred in other U.S. regions (6, 14). The lack of recent links between Floridian sequences and those from other U.S. states and international cases suggests an epidemic independently evolving from external influences. Yet, the uncertainty surrounding the time of cluster origin could indicate that epidemiological links among the sampled individuals are missing and that the large clusters may only be revealing a portion of even larger networks. Nevertheless, the detected clusters included exclusively Floridian strains suggesting that for the past several years, the Florida epidemic has been mainly driven by within state transmission rather than frequent outside introductions.

Overall, only 18% of HIV-1 subtype B sequences in Florida were linked in our study, which is comparable with the 22.1% clustering observed in New York City with similar sequence completeness (9). A study in Washington reported a similar clustering rate of 18% among prevalent infections with 49% sequence completeness (15). Yet, our linkage rate is much lower than studies conducted in other states, including North Carolina (50%) using a phylogeny-based approach, and Washington (46%) and Michigan (54%) using identical genetic distance-based methods but with high sequence completeness (16, 17). Although the proportion clustered in our study increased to 32% after removing individuals diagnosed before 2010, these findings were still lower than expected for the large number of sequences analyzed. Compared with simulations by Dasgupta et al., the low level of clustering observed in our study implies that only about 15% of PWH diagnosed between 2012 and 2017 in Florida have received a genotype (17). Yet, our data show that 44% received a genotype during this period (Table 1) and 41.3% received a genotype within 12 months of diagnosis (Table S1). Therefore, the large number of unlinked sequences is likely indicative of issues related to data completeness, rather than slowed transmission (18). While sequence completeness in Florida has improved, it is still below the CDC recommended rate of ≥60%. Despite the fact that molecular epidemiologic inferences are sensitive to data completeness and cannot account for undiagnosed infections (17), the results still provide actionable public health information for health officials (19).

The populations with the lowest odds of clustering in Florida were those with older diagnoses, living in a rural county, and female and Black PWH. These differences may be indicative of disparities in genotype coverage in these vulnerable groups. Cluster size was inversely associated with the age of cluster members—with a greater prevalence of younger PWH detected in the largest clusters. A similar trend was observed in North Carolina (16) and may be due to younger people having more recent diagnoses which increases the likelihood of capturing linkages. Our results are consistent with the epidemiological characteristics of the most at-risk groups for HIV infection in Florida (10). The lack of clustering among women with HIV-1 warrants further research, however, as Florida has the second highest number of women diagnosed with HIV in the nation as of 2017 (1, 2). Persons with mother-to-child (MTC) transmission had lower odds of clustering, which may indicate low rates of genotyping among pregnant women living with HIV, despite engagement in the health care system. Hence, genotyping among viremic pregnant women should be recommended. The reduced odds of clustering among Black PWH who accounted for the largest proportion (42%) of new HIV diagnoses in Florida in 2017 (10) is concerning, and likely a result of receiving suboptimal care. Lower odds of clustering among Black PWH has been observed in previous transmission cluster studies in the United States and may be linked to older or delayed diagnoses, or less genotypic drug resistance testing in this population (5, 9). Our assortative analysis is consistent with prior literature (20). Black PWH make up one of the largest percentages of undiagnosed PWH in the country and are more likely to have lower viral suppression (20, 21). In Florida, Black PWH are least likely to initiate care and have higher odds of drug resistance compared with White and Hispanic/Latino PWH (22). Persons living in rural counties also had lower odds of clustering. Clusters were highly assortative by geography, implying that the missing genetic links are living in the same geographic regions. Southern U.S. states have the highest rates of new HIV infections in nonmetropolitan areas as of 2018 (23). Almost half of PWH in priority clusters in 19 states, including 10 in the south, were not in EHE counties in a 2021 study (24). Recent outbreaks in rural areas driven by the opioid crisis highlight the increased risk for HIV transmission in rural America (25). Several barriers exist in rural communities for HIV prevention and care, including prolonged poverty, stigma, and lack of transportation, which may have contributed to the low clustering we observed in these populations (26). It is important to enhance outreach and public health efforts to help lessen the burden of infection among these groups.

This study revealed significant undersampling in key, possibly vulnerable, populations leading to more than expected unclustered sequences. Undiagnosed infections, lack of health care coverage, distrust in health care systems, HIV criminalization laws, and provider refusal may be among the reasons for decreased genotype testing in these populations. Restrictions on data sharing between states prevented the ability to investigate the degree to which interstate transmission is occurring. However, the CDC notifies states if there are rapidly growing clusters that have members from other states observed, because they have the deidentified data for all jurisdictions. States have their own reporting and data sharing laws, and not all states have implemented molecular HIV surveillance activities. In 2018, the CDC released the notice of funding opportunity, “PS-18-1802 - Integrated Prevention and Surveillance for Health Departments,” which paved the way to improve and increase molecular HIV surveillance activities across funded jurisdictions (27). Departments of Health across the country and the CDC could consider implementing strategies to increase genotyping from providers, while also working to address barriers to testing, and having conversations with the community to address privacy and ethical concerns.

Our phylogeographic analyses show that the Florida epidemic has been largely driven by within-state transmission and that most of the detected clusters have been well established in Florida for a relatively long time, suggesting that missing sequences are likely from Floridian PWH who are undiagnosed, out of care, or whose providers did not order a genotype test. Given the high rates of tourism across the state, it is possible that links to external introductions might missing due to the unavailability of sequences for the vacationers, or to the high proportion of unclustered individuals. The EHE plan prioritizes seven urban Florida counties for heightened HIV prevention services (3). These counties represented significant transmission hot spots in our study, and therefore, our findings support this approach. However, our study also highlights how phylogenetic analysis can provide information on health disparities that needs to be addressed. Our findings revealed low clustering frequency in vulnerable populations which may hinder the success of EHE and further widen disparities in access to HIV care and preventive services. The demographic diversity of PWH in the United States and the disproportionate epidemic among Black PWH necessitates approaches that are both equitable and tailored to key populations (28). Further, HIV transmission is not limited to high incidence areas but can result from influx and efflux of infections to and from these locations limiting success of geographically focused interventions (29). Thus, directing resources to rural Florida counties, in addition to women and Black PWH, will be important to achieve the EHE goals.

When performing our analyses, we considered the ethical discussions recently raised by Tordoff et al. (30), including the inference of transmission directionality among individuals and vulnerable populations, and assortativity of transmission categories. To this effect, our analyses were careful not to infer any individual- or demographic (age, gender, and race/ethnicity) group-level transmission directionality and we exclusively reported virus flow across large geographic regions (i.e., counties) rather than individual groups. Cluster analysis included both geographic and demographic strata, and we focused on differences among clustered and unclustered sets. The findings confirm structural disparity, but also pose new research questions, such as the lack of linkage among women. In the assortativity analysis, we elected to report only spatiotemporal and nonspecific cross-demographic ranges. We acknowledge the lack of theory on how phylogenetic-derived indices are influenced by structural causes of HIV disparity, and that the understanding of such causal pathways at both individual- and community-level is critical to design better interventions. Nonetheless, one of the EHE operational pillars is geographic prioritization, and our objective was to confirm if the current set of Florida counties should be reconsidered. Our findings are of great public health utility as they provide the evidence needed to reconsider additional counties in future iterations of the EHE, with beneficence to the population, to ultimately help achieve health equity and reach vulnerable populations more effectively. In the context of HIV stigma and criminalization, we recognize that there is a need to conduct in parallel ethical discussions on the usage of molecular surveillance data to reduce any potential direct harm to individuals or reiteration of systemic discrimination, and to learn more about the concerns of the community.

### Conclusion.

Our study is the most comprehensive analysis of HIV-1 transmission inferred from sequence data in Florida to date. We revealed the presence of many large clusters in a background of low clustering frequency despite sufficient sampling density, resulting in most infections being unlinked. Evaluation of potential linkages to external sequences from public databases did not yield significant improvement in clustering. Significant health disparities were observed. Individuals living in rural counties, women, and Black PWH were the least likely to cluster in this study and represent subpopulations in whom EHE interventions should also be prioritized. Transmission patterns also showed that while the seven urban counties identified as focus regions for Florida are justifiable targets for the initial phase of the EHE plan, consideration of additional counties, both suburban and rural, and enhanced focus on key populations will be important for achieving EHE goals in Florida.

## MATERIALS AND METHODS

### Ethics statement.

The study protocol was approved by the University of Florida’s Institutional Review Board (IRB) #IRB201901041 (extending #IRB201703199) and FDoH IRB protocol #2020-069 as exempt. We received sequence data and metadata from FDOH in fully deidentified format according to HIPAA regulations. The study data are not available in any public repository; however, for replication purposes, a request to the FDOH can be made following state and federal regulations and compliance to all required ethical and privacy policies (https://www.cdc.gov/hiv/pdf/funding/announcements/ps18-1802/cdc-hiv-sequence-guidance.pdf). Request are independently reviewed by FDOH.

### Sequence data and molecular transmission network analysis.

Partial *pol* sequences (*N* = 34,446) for diagnosed PWH who received HIV-1 genotyping during 2007 to 2017 were retrieved from the FDOH. Molecular network analyses were restricted to years 2012 to 2017, to reflect the updated state guidelines on molecular surveillance that led to increased sampling and reporting during this period, including reference sequences from Los Alamos National Laboratory database (https://www.hiv.lanl.gov). Molecular networks were constructed using MicrobeTrace (31). Bayesian phylogeographic analysis was performed in BEAST (32) using an asymmetric substitution model for discrete traits (i.e., locations) with Bayesian stochastic search variable selection, an uncorrelated relaxed clock and the Skyline tree topology prior (see Supplementary Methods for details). Infection rates (estimated as the number of persons in the cluster minus 1, divided by the total person-time living with HIV in the cluster, i.e., the time between the inferred date of infection for each person in the cluster and the end of the period of observation, during which these persons could have contributed toward new infection events) were calculated for the largest clusters using the node age estimates from BEAST as previously done by Oster et al. (8) (see Supplementary Methods for details). Xmls and scripts are available at https://github.com/cmavian/HIV-Florida-paper.

### Statistical analysis.

De-identified demographic and diagnosis data were obtained from the FDOH’s eHARS. Counties were coded into districts (central east and west, northeast, northwest, southeast and southwest) and by urban versus rural designation, using the 2010 U.S. Census. Demographic and clinical characteristics were compared according to cluster status (clustered versus unclustered) and, among those who clustered, by cluster size. Multivariable main-effects logistic regression models were fitted to associate participant characteristics with cluster status. A sensitivity analysis removing PWH diagnosed prior to 2010 was performed to compare the percentage of sequences that clustered and the correlates of clustering in more recently diagnosed PWH (see Supplementary Methods for details). Scripts are available at https://github.com/cmavian/HIV-Florida-paper.
