# Supplementary material for: Molecular Epidemiology of HIV-1 Subtype B Infection across Florida Reveals Few Large Superclusters with Metropolitan Origin
Source: Microbiol Spectr. 2022 Oct 12;10(6):e01889-22. doi: 10.1128/spectrum.01889-22 (PMC9769514; doi:10.1128/spectrum.01889-22)
Supplement: Supplemental file 1 — Supplemental material. Download spectrum.01889-22-s0001.pdf, PDF file, 1.1 MB [file spectrum.01889-22-s0001.pdf]

Phylodynamic characterization of HIV-1 subtype B transmission across Florida reveals few large superclusters with Metropolitan origin.

Supplementary Methods

Data source/availability

The study protocol was approved by the University of Florida's Institutional Review Board (IRB) #IRB201703199 and by FDOH's IRB as exempt, as all data were collected per routine HIV surveillance and this work was conducted for public health purposes. The data source is the FDOH's enhanced HIV/AIDS Reporting System (eHARS), which is made available by applying for research use of de-identified surveillance data to the FDOH Bureau of Communicable Diseases. This manuscript adheres to the Strengthening the Reporting of Molecular Epidemiology for Infectious Diseases statement on responsible reporting.

Sequence data

We obtained 34,446 partial *pol* sequences, encompassing protease and part of reverse transcriptase from the FDOH for diagnosed PWH who received HIV-1 genotyping during 2007-2017. In cases of multiple sequences per person, we included only the earliest sequence. Transmission network analyses were restricted to years 2012-2017 to reflect the updated state guidelines on molecular surveillance that led to increased sampling and reporting during this period. Sequences were subtyped using the COntext-based Modeling for Expeditious Typing subtyping tool and only subtype B sequences were retained for the current analysis (1). Drug resistance mutation sites were stripped from the alignment prior to phylogenetic analysis. To account for potential imported infections, we supplemented our dataset with external sequences from Los Alamos National Laboratory (LANL) database (https://www.hiv.lanl.gov) by

performing a nucleotide BLAST search against the global HIV-1 subtype B sequences, discarding duplicates. This analysis was repeated with 21 random samples of unclustered Florida sequences (each sample set containing 100 Florida sequences to represent approximately 10% of the total unclustered population) and the LANL global subtype B sequences to detect potential external linkages to the local unclustered population.

Covariates

De-identified demographic and HIV diagnosis data were obtained from the FDOH's eHARS. We collected data on age at genotype collection, birth sex, race/ethnicity (Black/African American (hereafter referred to as Black), White, Hispanic/Latino, and Other [American Indian/Alaska Native, Asian, Native Hawaiian/Pacific Islander, or Multi-race]), county of residence, birth country, transmission category (heterosexual contact [HET], intravenous drug use [IDU], men who have sex with men [MSM], mother-to-child [MTC], or other/unknown), and diagnosis and genotype specimen collection (sampling) years. For individuals with two or more reported transmission categories (occurring for IDU, MSM, and HET), we assigned the category for which the transmission probability is highest (IDU > MSM > HET). Counties were coded into districts (central east and west, northeast, northwest, southeast and south west) as defined by the Florida Local Government Information Systems Association and by urban versus rural designation, as defined by the 2010 US Census. Birth countries were coded into regions [North America vs. outside of North America (Africa, Asia Pacific, Caribbean, Europe, Latin America)] for analysis.

Molecular transmission network

Molecular transmission networks were constructed using MicrobeTrace (2), a bioinformatics software tool that securely integrates sequence and epidemiologic data to detect and visualize transmission networks. MicrobeTrace uses the Tamura-Nei 1993 nucleotide substitution model to compute pairwise genetic distances between nucleotide sequences. We used a 1.5% genetic distance threshold (0.015 nucleotide substitutions per site representing approximately 7-8 years of viral evolution) for cluster detection. To reduce the computational power needed to analyze this large dataset, the final dataset was subset into four smaller datasets according to cluster size (2-4, 5-10, 11-28, 29-70 sequences). Associated epidemiologic data were integrated in MicrobeTrace for visualization and exploration of the inferred transmission networks.

Statistical analysis

Demographic and clinical characteristics of PWH were compared according to cluster status (clustered versus unclustered) and, among those who clustered, by cluster size. Descriptive statistics were calculated to ascertain baseline group differences. Multivariable main-effects logistic regression models were fitted to associate demographic and clinical characteristics with cluster status using two feature selection approaches, bidirectional stepwise selection and boosting (centered and non-centered) with component-wide univariate linear models using the R package 'mboost.' A sensitivity analysis removing PWH diagnosed prior to 2010 was performed to compare the percentage of sequences that clustered and the correlates of clustering in more recently diagnosed PWH. To assess whether the proportion of sequences that clustered by county was dependent on the number of sequences available for the analysis, adjusting for local population prevalence of HIV, we fit a bivariate linear regression model to the data. In the analysis of cluster features, we evaluated whether PWH tend to link with other PWH who share a

common attribute, referred to as assortative mixing in network analyses (3). Assortativity coefficients were generated using the R package ‘igraph.’ All statistical analyses were conducted in R, version 3.6.1.

Bayesian phylogeography

Bayesian phylogeographic analysis was performed to assess transmission of HIV-1 within Florida by region (north, central, and south) for the nine largest clusters identified by MicrobeTrace (each containing ≥29 sequences). Prior to performing the Bayesian analyses, we assessed the quality of the phylogenetic signal present in the datasets using DAMBE7 (9), IQ-TREE (5) and TempEst (6) to check for the absence of nucleotide substitution saturation (Figure S1a), and the presence of phylogenetic (Figure S1b) and molecular clock signals, respectively (Figure S1c). Additionally, we compared the rate estimate of each of the largest trees with the expectation in the absence of temporal structure by adding an operator that permutes the tip dates. After confirming the conditions were met, we performed the phylogeographic tree search with the Bayesian Evolutionary Analysis by Sampling Trees (BEAST) v1.10.4 software (7). BEAST was run using the Hasegawa-Kishino-Yano nucleotide substitution model with empirical base frequencies and gamma distribution of site-specific rate heterogeneity, an asymmetric substitution model for discrete traits (i.e. locations) with Bayesian stochastic search variable selection (BSSVS), an uncorrelated relaxed clock and the Skyline tree topology prior (8). A molecular clock rate prior of 1.47E-3 substitutions/site was applied as this was the average clock rate for the trees with the strongest phylogenetic and temporal signal and is consistent with prior studies using HIV-1 *pol* sequences (9). Markov Chain Monte Carlo samplers were run for 200-250 million generations. Adequate mixing was assessed by calculating the effective sampling size (ESS) of parameter estimates (cutoff ESS ≥250). The maximum clade credibility (MCC)

summary tree was merged with geographic coordinates for each Florida region to identify well-supported pairwise diffusion rates between regions (summarized with Bayes factor [BF]) in SpreaD3 (10). BF values between 3 and 20 are generally accepted as probable ("positive support") evidence of phylogeographic diffusion between regions; values above 20 are generally considered as strong support (11). Adjusted BF (aBF) values were estimated by performing an additional migration analysis with BSSVS, the "tip-state-swap" analysis (12), during which the sequence traits (i.e. location) are randomly permuted during the MCMC integration to account for sampling bias in the phylogeographic analysis. BF adjusted values were calculated with a suite of in-house R scripts. The MCC trees were inferred from the posterior distribution of trees using TreeAnnotator v1.10.4, specifying a burn-in of 10% and median node heights, then edited graphically in FigTree v1.4.4 (http://tree.bio.ed.ac.uk/software/figtree/) and with 'ggtree' implemented in R. Transmission rates were calculated for the largest clusters using the node age estimates from BEAST as previously done by Oster *et al.* (13):

$$Transmission\ rate = \frac{(\text{Total no. of cluster members} - 1)}{[\Sigma(\text{node ages}) + \text{longest node age}]}$$

References

1. Struck D, Lawyer G, Ternes AM, Schmit JC, Bercoff DP. COMET: adaptive context-based modeling for ultrafast HIV-1 subtype identification. Nucleic Acids Research. 2014 Oct 13;42(18):e144–e144.

2. Campbell EM, Boyles A, Shankar A, Kim J, Knyazev S, Cintron R, et al. MicrobeTrace: Retooling molecular epidemiology for rapid public health response. PLoS Comput Biol. 2021 Sep 7;17(9):e1009300.

3. Newman MEJ. Mixing patterns in networks. Physical Review E [Internet]. 2003 Feb 27 [cited 2019 Nov 20];67(2). Available from: https://link.aps.org/doi/10.1103/PhysRevE.67.026126

4. Xia X. DAMBE7: New and Improved Tools for Data Analysis in Molecular Biology and Evolution. Kumar S, editor. Molecular Biology and Evolution. 2018 Jun 1;35(6):1550–2.

5. Nguyen LT, Schmidt HA, von Haeseler A, Minh BQ. IQ-TREE: A Fast and Effective Stochastic Algorithm for Estimating Maximum-Likelihood Phylogenies. Molecular Biology and Evolution. 2015 Jan;32(1):268–74.

6. Rambaut A, Lam TT, Max Carvalho L, Pybus OG. Exploring the temporal structure of heterochronous sequences using TempEst (formerly Path-O-Gen). Virus Evol. 2016 Jan;2(1):vew007.

7. Drummond AJ, Suchard MA, Xie D, Rambaut A. Bayesian Phylogenetics with BEAUti and the BEAST 1.7. Molecular Biology and Evolution. 2012 Aug;29(8):1969–73.

8. Hall MD, Woolhouse MEJ, Rambaut A. The effects of sampling strategy on the quality of reconstruction of viral population dynamics using Bayesian skyline family coalescent methods: A simulation study. Virus Evolution [Internet]. 2016 Jan [cited 2019 Dec 10];2(1). Available from: http://academic.oup.com/ve/article/doi/10.1093/vew003/1753436/The-effects-of-sampling-strategy-on-the-quality-of

9. Leitner T, Albert J. The molecular clock of HIV-1 unveiled through analysis of a known transmission history. Proceedings of the National Academy of Sciences. 1999 Sep 14;96(19):10752–7.

10. Bielejec F, Baele G, Vrancken B, Suchard MA, Rambaut A, Lemey P. SpreaD3: Interactive Visualization of Spatiotemporal History and Trait Evolutionary Processes. Molecular Biology and Evolution. 2016 Aug;33(8):2167–9.

11. Bielejec F, Rambaut A, Suchard MA, Lemey P. SPREAD: spatial phylogenetic reconstruction of evolutionary dynamics. Bioinformatics. 2011 Oct 15;27(20):2910–2.

12. Edwards CJ, Suchard MA, Lemey P, Welch JJ, Barnes I, Fulton TL, et al. Ancient Hybridization and an Irish Origin for the Modern Polar Bear Matriline. Current Biology. 2011 Aug;21(15):1251–8.

13. Oster AM, France AM, Panneer N, Bañez Ocfemia MC, Campbell E, Dasgupta S, et al. Identifying Clusters of Recent and Rapid HIV Transmission Through Analysis of Molecular Surveillance Data: JAIDS Journal of Acquired Immune Deficiency Syndromes. 2018 Dec;79(5):543–50.
